# A Longitudinal Study Reveals Metabolomic Markers for Individuals at Risk, Disease Severity, and Treatment Response in Rheumatoid Arthritis

**DOI:** 10.1002/advs.202504414

**Published:** 2025-08-13

**Authors:** Chenxi Zhu, Jiayi Xu, Siyu He, Qian Niu, Yi Liu, Xin Guo, Huifang Hu, Rui Sun, Tao Chen, Yan Liu, Zhiqiang Xu, Na Jiang, Lipu Yao, Lunzhi Dai, Qinghua Zou, Fanxin Zeng, Liesu Meng, Inger Gjertsson, Rikard Holmdahl, Bin Yang, Dan Du, Yi Zhao

**Affiliations:** ^1^ Department of Rheumatology and Immunology Clinical Institute of Inflammation and Immunology Frontiers Science Center for Disease‐related Molecular Network West China Hospital Sichuan University Chengdu Sichuan 610041 P. R. China; ^2^ National Clinical Research Center for Geriatrics Department of General Practice State Key Laboratory of Biotherapy West China Hospital Sichuan University Chengdu Sichuan 610041 P. R. China; ^3^ Department of Laboratory Medicine West China Hospital, Sichuan University Chengdu Sichuan 610041 P. R. China; ^4^ Advanced Mass Spectrometry Center and Research Core Facility Frontiers Science Center for Disease‐related Molecular Network West China Hospital, Sichuan University Chengdu Sichuan 610213 P. R. China; ^5^ Department of Rheumatology and Immunology First Affiliated Hospital of Army Military Medical University Chongqing 400038 P. R. China; ^6^ Department of Big Data and Biomedical AI College of Future Technology Peking University Beijing 100871 P .R. China; ^7^ Department of Clinical Research Center Dazhou Central Hospital Dazhou Sichuan 635000 P. R. China; ^8^ Institute of Basic Medicine and Forensic Medicine North Sichuan Medical College Nanchong Sichuan 637000 P. R. China; ^9^ National‐Local Joint Engineering Research Center of Biodiagnostics and Biotherapy Second Affiliated Hospital Xi'an Jiaotong University Xi'an Shaanxi 710004 P. R. China; ^10^ Department of Rheumatology and Inflammation Research Institute for Medicine Sahlgrenska Academy University of Gothenburg Gothenburg 40530 Sweden; ^11^ Medical Inflammation Research Section of Immunology Research Department of Medical Biochemistry and Biophysics Karolinska Institute Stockholm 17177 Sweden

**Keywords:** longitudinal profiling, machine learning, metabolomics, rheumatoid arthritis, therapy response

## Abstract

Rheumatoid arthritis (RA) is a systemic inflammatory joint disease characterized by heterogeneous clinical manifestations, which requires deeper exploration in identifying reliable biomarkers for early diagnosis, monitoring, and treatment assessment. The aim is to discover plasma metabolomic markers to predict RA onset, assess disease activity, and forecast treatment efficacy. The study includes 209 established RA patients who are disease‐modifying antirheumatic drugs‐free for six months prior to enrollment, with 197 of them followed for 3–6 months to assess treatment response. Additionally, 56 individuals at risk are recruited, with 34 completing a 5–7‐year follow‐up. Analysis reveals that metabolites related to methylation and redox imbalance, such as S‐adenosylmethionine, sarcosine, nicotinamide adenine dinucleotide, glutathione, etc., are associated with RA development and severity, and contribute to its heterogeneity across age, sex, and anti‐citrullinated protein autoantibody status. Ridge regression models are constructed using metabolite and clinical features for the response to methotrexate (MTX) plus leflunomide, achieving an average receiver operating characteristic (ROC) score of 0.83, and for the MTX plus hydroxychloroquine, achieving an average ROC score of 0.92. In conclusion, our findings reveal RA metabolomic alterations, aiding early diagnosis and treatment response.

## Graphical abstract

This study investigates plasma metabolomic biomarkers for rheumatoid arthritis (RA) development using longitudinal data from 209 RA patients and 56 individuals at risk. It also reveals metabolic signatures linked to severity, age, sex, and anti‐citrullinated protein autoantibody in RA. Machine learning models integrates metabolic and clinical features to predict therapy response. Findings offer insights into precision diagnostics and tailored interventions. 

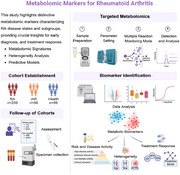

## 1 Introduction

Rheumatoid arthritis (RA) represents a chronic systemic inflammatory disorders predominantly impacting joint structures.^[^ ^1^ ^]^ Despite advancements in therapeutic strategies, significant challenges persist in early diagnosis, accurate prognosis, and personalized treatment selection due to their distinct manifestations across stages.^[^ ^2^ ^]^ Current clinical biomarkers, such as rheumatoid factor and anti‐citrullinated protein autoantibody (ACPA), lack sufficient sensitivity and specificity to predict disease onset, stratify severity, and forecast therapeutic outcomes.^[^ ^3^ ^]^ Furthermore, the heterogeneous nature of RA pathophysiology underscores the urgent need for novel biomarkers that reflect dynamic molecular changes across disease stages and therapeutic interventions.

The management of RA is anchored on a structured approach known as treat‐to‐target, where conventional synthetic disease‐modifying antirheumatic drugs (csDMARDs) are central.^[^ ^4^ ^]^ Despite this, a significant portion of patients (30–60%) display unsatisfactory responses to csDMARDs, posing a challenge.^[^ ^5^ ^]^ Various clinical parameters or laboratory measurements have been explored for their predictive value regarding csDMARDs responses, and omics strategies have been utilized to provide potential predictive molecular features.^[^ ^6^, ^7^, ^8^, ^9^, ^10^ ^]^

Metabolomics has recently emerged as a potent tool for identifying disease‐specific metabolic signatures, gaining prominence for its ability to capture global biochemical perturbations in biological systems.^[^ ^11^ ^]^ Unlike genomic or proteomic profiles, metabolites serve as direct functional readouts of cellular activity, offering insights into dysregulated pathways in RA.^[^ ^12^ ^]^ Recent cross‐sectional studies have identified potential metabolomic markers associated with RA pathogenesis; however, their findings are often constrained by static sampling designs, limited sample sizes, lack of longitudinal follow‐up, and insufficient control over pre‐enrollment medication use.^[^ ^13^ ^]^ Therefore, well‐designed longitudinal metabolomic studies are crucial for capturing meaningful metabolic trajectories and identifying stable biomarkers indicative of disease development and treatment outcomes.

This longitudinal metabolomic study tracked healthy individuals, at‐risk individuals (IAR), and untreated RA patients (both ACPA positive and negative) using LC‐MS/MS ‐based targeted profiling. We mapped dynamic plasma metabolic trajectories across the RA continuum—from preclinical stages to treated disease, revealing significant associations with disease activity, demographics, and ACPA status. Follow‐up data from csDMARDs‐treated patients revealed therapy‐responsive metabolic pathway remodeling and predictive biomarkers for methotrexate (MTX), leflunomide (LEF), and hydroxychloroquine (HCQ) combination efficacy. These findings advance mechanistic understanding of RA development and demonstrate the translational potential of longitudinal metabolomics for precision management in autoimmune diseases, enabling early diagnosis, tailored therapies, and improved outcomes.

## 2 Results

### 2.1 Metabolomic Profiling of Plasma Samples from Healthy Individuals, IAR, and Established RA Patients

**Figure 1:**
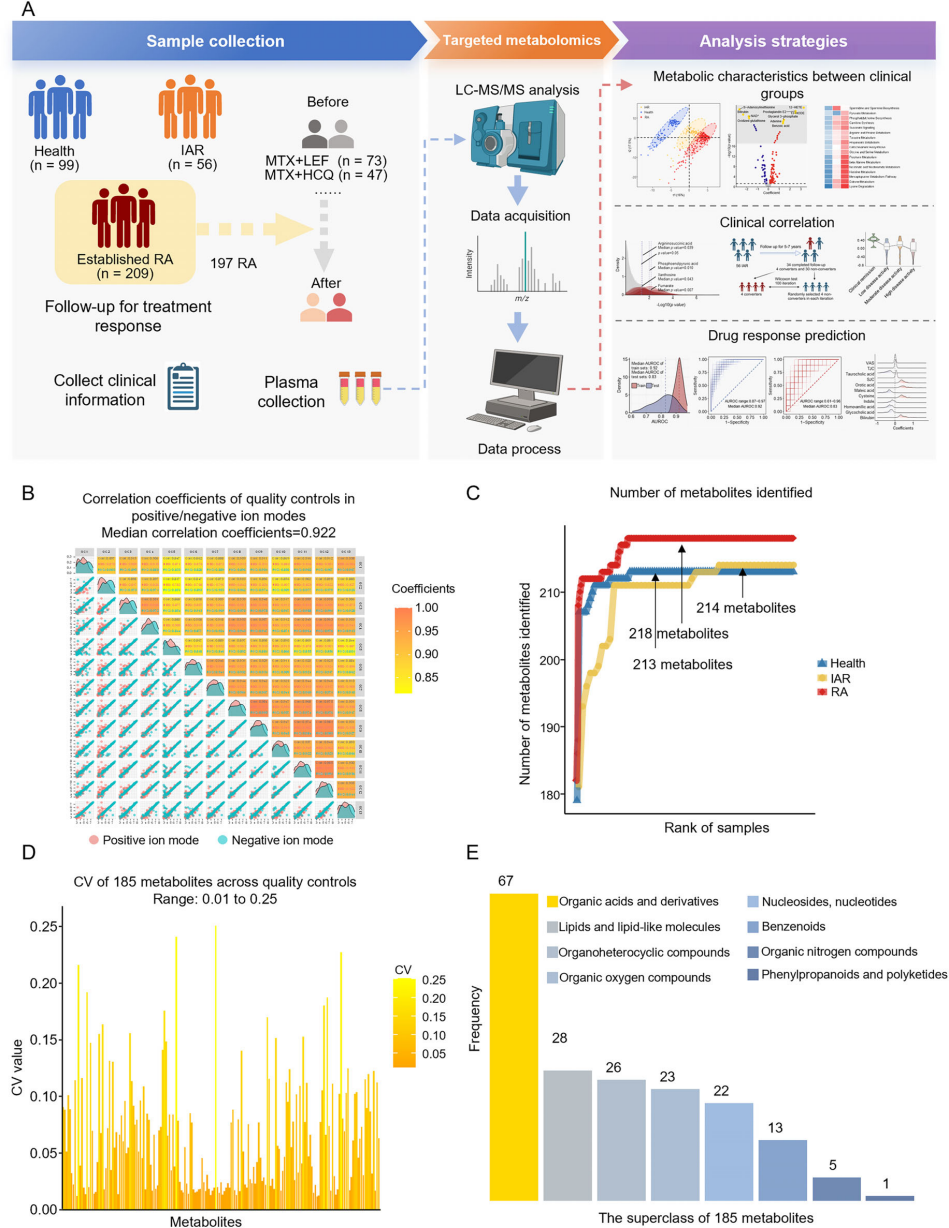
Metabolomic analysis workflow and quality control analysis. A) Schematic illustration of the plasma metabolomics workflow, including sample collection, targeted metabolomic detection, and data analyses. B) Spearman's correlation coefficients of quality controls (QCs) samples in both positive (red) and negative (blue) ion modes. The upper panels show color‐coded pairwise correlation values between QCs; the lower panels display pairwise scatter plots. Density curves of each QCs are shown along the diagonal. C) The cumulative count of metabolites identified across health (blue, *n* = 99), individuals at risk (IAR) (yellow, *n* = 56), and rheumatoid arthritis (RA) groups (red, *n* = 209). D) Coefficient of variation (CV) values for 185 metabolites across QCs samples. E) Classification and distribution of the 185 metabolites by superclass. Classification is annotated using the Human Metabolome Database.

In this study, 209 patients diagnosed with RA from West China Hospital were recruited (**Figure** **1**A; Table S1A, Supporting Information). The cohort predominantly consisted of female patients (174, representing 83.3%). The mean age was 51 years old, spanning from 16 to 77. The disease activity score in 28 joints using C‐reactive protein (DAS28‐CRP) ranged from 1.40 to 8.39, averaging 3.66. Patients negative for ACPA presented an older average age of 56 years (compared to 51 years for ACPA‐positive counterparts) and demonstrated higher CRP, averaging 23.97 (versus 14.17 for ACPA‐positive RA), as detailed in **Table** **1**. Eligibility for the study required that participants had not been treated with any disease‐modifying antirheumatic drugs (DMARDs) for a minimum of six months before sample collection. 197 RA patients were followed for three to six months after treatment with MTX monotherapy or a combination of csDMARDs (Table S1A, Supporting Information). During this period, clinical data and blood specimens were gathered at the conclusion. Furthermore, the study included healthy individuals and IAR for comparative purposes. Among the 99 healthy individuals, the mean age was 50 years, ranging from 38 to 76 years (comprising 79 females and 20 males). The IAR group included 56 individuals with a mean age of 50 years, age distribution from 29 to 74 years (30 females and 26 males) (Table 1).

**Table 1:** Clinical attributes of the research participants.

Baseline Clinical Information	All Patients (N = 209)	ACPA status			IAR (N = 56)	Health (N = 99)
		Positive (N = 183)	Negative (N = 14)	Missing (N = 12)		
Female, n (%)	174 (83.3%)	151 (82.5%)	12 (85.7%)	11 (91.7%)	30 (53.6%)	79 (79.8%)
Age, years	51 (16‐77)	51 (16‐75)	56 (28‐77)	50(16‐76)	50 (29‐74)	50 (38‐76)
Body Mass Index(kg/m^2^)	22.54 (14.38‐38.06)	22.54 (15.94‐38.06)	22.43 (14.38‐25.85)	22.57 (15.63‐28.20)	22.34 (17.43‐29.07)	22.67 (17.53‐38.53)
Time from symptom onset to diagnosis, months	25 (0‐420)	27 (0‐420)	15 (6‐48)	15 (6‐84)	–	–
Time from diagnosis to sampling, months	64 (0‐492)	63 (0‐492)	47 (0‐156)	85 (0‐228)	–	–
Duration, months^a)^	89 (6‐498)	90 (6‐498)	62 (10‐204)	100 (6‐234)	–	–
DAS28‐CRP	3.66 (1.40‐8.39)	3.69 (1.40‐8.39)	3.67 (1.71‐5.53)	3.19 (1.78‐5.76)	–	–
Disease Activity Level^b)^, n (%)					–	–
Clinical Remission	41 (19.6%)	34 (18.6%)	2 (14.3%)	5 (41.7%)	–	–
Low Disease Activity	45 (21.5%)	39 (21.3%)	4 (28.6%)	2 (16.7%)	–	–
Moderate Disease Activity	95 (45.5%)	86 (47.0%)	6 (42.9%)	3 (25.0%)	–	–
High Disease Activity	28 (13.4%)	24 (13.1%)	2 (14.3%)	2 (16.7%)	–	–
CRP (mg/L)	14.30 (1.00‐197.00)	14.17 (1.00‐197.00)	23.97 (2.00‐134.00)	5.14 (1.00‐12.00)	–	–
Tender Joints In 28‐Joint Count	5 (0‐28)	6 (0‐28)	5 (0‐24)	4 (0‐16)	–	–
Swollen Joints In 28‐Joint Count	2 (0‐28)	2 (0‐28)	3(0‐22)	2 (0‐6)	–	–
Visual Analog Scale	45.13 (0‐100)	45.74 (0‐100)	44.29 (20‐70)	36.83 (2‐90)	–	–
Health Assessment Questionnaire	8.11 (0‐80)	8.08 (0‐80)	10.21 (0‐41)	6.08 (0‐24)	–	–
Smokers, n (%)	17 (8.1%)	16 (8.7%)	0 (0%)	1 (8.3%)	13 (23.2%)	8 (8.0%)
With comorbidity, n (%)						
Hypertension	35 (16.7%)	28 (15.3%)	4 (28.5%)	3 (25%)	4 (7.1%)	11 (11.1%)
Hyperlipidemia	9 (4.3%)	7 (3.8%)	0 (0%)	2 (16.6%)	5 (8.9%)	1 (1.0%)
Hyperglycemia	12 (5.7%)	9 (4.9%)	2 (14.2%)	1 (8.3%)	6 (10.7%)	6 (6.0%)

*Note*: Continuous variables are presented as mean values (range), and categorical variables are displayed as counts (percentages).

^a)^ Duration: the length of time the patient has had symptoms/disease, not the length of time since RA diagnosis. Symptom onset was defined as the time of onset of inflammatory joint pain and/or early morning stiffness and/or joint‐related soft tissue swelling;

^b)^ Clinical remission: DAS28‐CRP < 2.6; Low disease activity: 2.6 ≤DAS28‐CRP ≤ 3.2; moderate disease activity: 3.2 < DAS28‐CRP ≤ 5.1; high disease activity: DAS28‐CRP > 5.1.

We then performed targeted metabolic analysis using 238 reference metabolite standards via multiple reaction monitoring (MRM) to detect water‐soluble metabolites in plasma samples.^[^ ^14^, ^15^, ^16^ ^]^ The median correlation coefficient of quality controls (QCs) across all batches was 0.922 (Figure 1B). Using the criterion of detecting at least one sample per clinical group, we ultimately identified 218 metabolites in the RA group (consisting of both baseline and follow‐up samples), 214 in the IAR, and 213 in the health group, and the sample size was sufficient to detect the maximum number of metabolites in each group (Figure 1C). We retained metabolites that had less than 80% missing values across all samples, resulting in a total of 185 metabolites for further analysis (Table S1B, Supporting Information). The coefficient of variation (CV) values of 185 metabolite abundances across QCs remained below 0.3 (Figure 1D). Regarding the superclass, 67 of these 185 metabolites were identified as organic acids and derivatives, 28 as lipids and lipid‐like molecules, and 26 as organoheterocyclic compounds (Figure 1E; Table S1C, Supporting Information).

### 2.2 Metabolic Variations Among Healthy Individuals, IAR, and Established RA Patients

**Figure 2:**
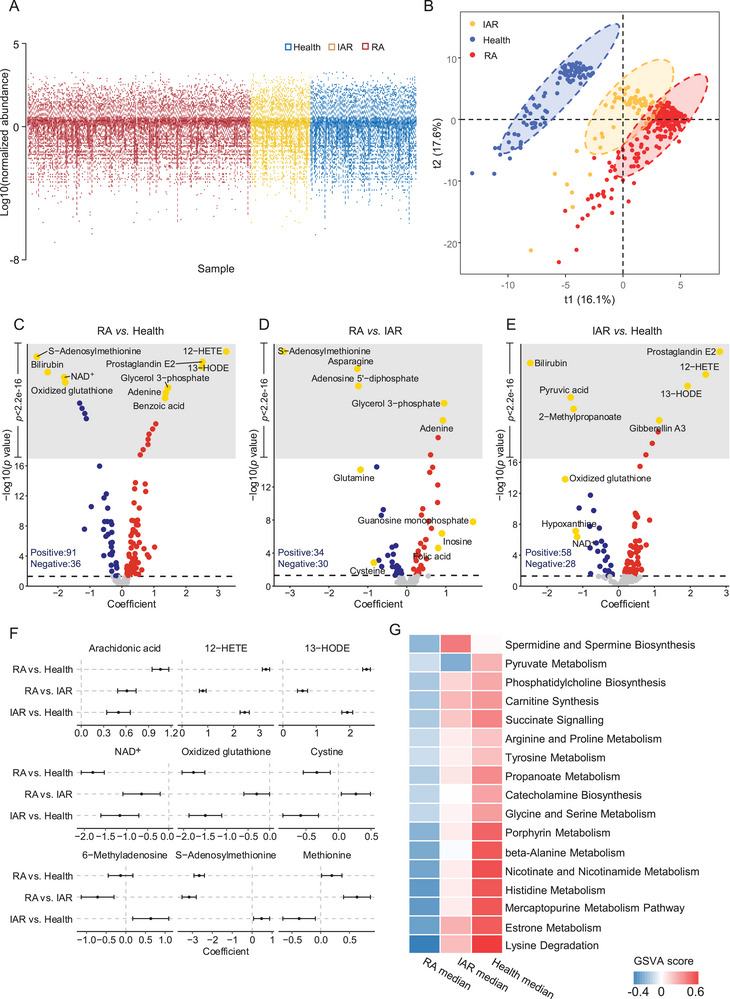
Plasma metabolomic differences among RA, IAR, and healthy individuals. A) Boxplots illustrating the median and interquartile range of normalized metabolites’ abundance (*n* = 185). B) The partial least squares discriminant analysis (PLS‐DA) scatter plot displaying the distribution of samples across baseline RA (red), IAR (yellow), and health (blue) on the first two principal components. C, D, E) Volcano plot showing metabolite associations across clinical groups (RA vs. health, RA vs. IAR, and IAR vs. health) via generalized estimating equation (GEE) model, adjusted for age, sex, body mass index (BMI), smoking status, and comorbidities. Metabolites with significant positive associations (coefficient > 0, *p* < 0.05, two‐sided *t*‐test) are shown in red; significant negative associations (coefficient < 0, *p* < 0.05, two‐sided *t*‐test) are shown in blue. The top 10 most significant metabolites are highlighted in gold. The gray shaded region represents metabolites with p‐values smaller than the minimum detectable threshold in R (2.2E‐16). F) Forest plot of regression coefficients (95% CI) for metabolites significantly altered in RA versus health and IAR versus health comparisons, involved in inflammation, methylation, or redox processes. G) Heatmap showing the median gene set variation analysis (GSVA) scores of differential pathways across three clinical groups. Significance was determined using GEE analysis (*p* < 0.05, two‐sided *t*‐test), adjusting for age, sex, BMI, smoking status, and comorbidities.

We compared metabolites in plasma from healthy, IAR, and those with established RA (209 baseline samples). The distribution of normalized abundance of metabolites across three groups was consistent, highlighting minimal batch effects (**Figure** **2**A). The partial least squares discriminant analysis (PLS‐DA) revealed clear separability among the three groups, with the IAR group showing closer proximity to the RA group than the health (Figure 2B). Using the generalized estimating equation (GEE) model, we examined the association between metabolite levels and clinical status, adjusting for age, sex, body mass index (BMI), smoking status, and common metabolism‐related comorbidities status, including hypertension, hyperglycemia, and hyperlipidemia (Figure 2C–E; Table S2A, Supporting Information). For the comparison between RA and health, 91 metabolites were positively associated with RA, while 36 showed negative associations. In the RA versus IAR comparison, 34 were positively correlated, and 30 were negatively correlated with RA. For IAR versus health, 58 were positively correlated, and 28 were negatively correlated. We identified altered levels of methylation‐related metabolites in RA, marked by a pronounced reduction in S‐adenosylmethionine (SAM), alongside elevated levels of other methyl carriers or donors such as folic acid, betaine, and methionine. Several novel metabolite patterns were found, including decreased hypoxanthine and increased levels of xanthine, histidine, threonine, and branched‐chain amino acids (BCAAs). (Table S2A, Supporting Information). In addition, we found that inflammation‐ and oxidative stress‐related metabolic alterations were already present during the preclinical phase. These included elevated pro‐inflammatory metabolites such as arachidonic acid (AA), and its derivatives 12‐HETE and 13‐HODE in IAR. In contrast, molecules involved in maintaining redox balance—nicotinamide adenine dinucleotide (NAD⁺), glutathione (GSH), oxidized glutathione (GSSG), and cystine (essential for GSH synthesis)—were reduced in IAR compared to healthy individuals. In addition, 6‐methyladenosine was specifically elevated in IAR, suggesting increased mRNA methylation during this stage (Figure 2F).

Beyond analyzing individual molecules, we conducted a GEE analysis to examine the correlations between the metabolic pathways and clinical groups. Each pathway's score was calculated by gene set variation analysis (GSVA) based on the small molecule pathway database (SMPDB).^[^ ^17^ ^]^ The significantly altered pathways were predominantly decreased in IAR and RA compared to health (Figure 2G; Table S2B, Supporting Information). These pathways included several amino acid metabolisms (e.g., lysine, histidine, glycine, etc.) and energy‐related cycles (e.g., nicotinate and nicotinamide metabolism, carnitine synthesis, and propanoate metabolism). Notably, nicotinate and nicotinamide metabolism indicated a mitochondrial defect under inflammatory conditions and contributing to the decreased levels of NAD^+^. Furthermore, we identified unique metabolic alterations in IAR: spermidine/spermine biosynthesis was elevated compared to both RA and health, while a reduction was observed in pyruvate metabolism. Monitoring these dynamic alterations at the preclinical stage may facilitate the prediction of disease development (Figure 2G).

### 2.3 Influences of Sex, Age, and ACPA Status on RA Metabolism

**Figure 3:**
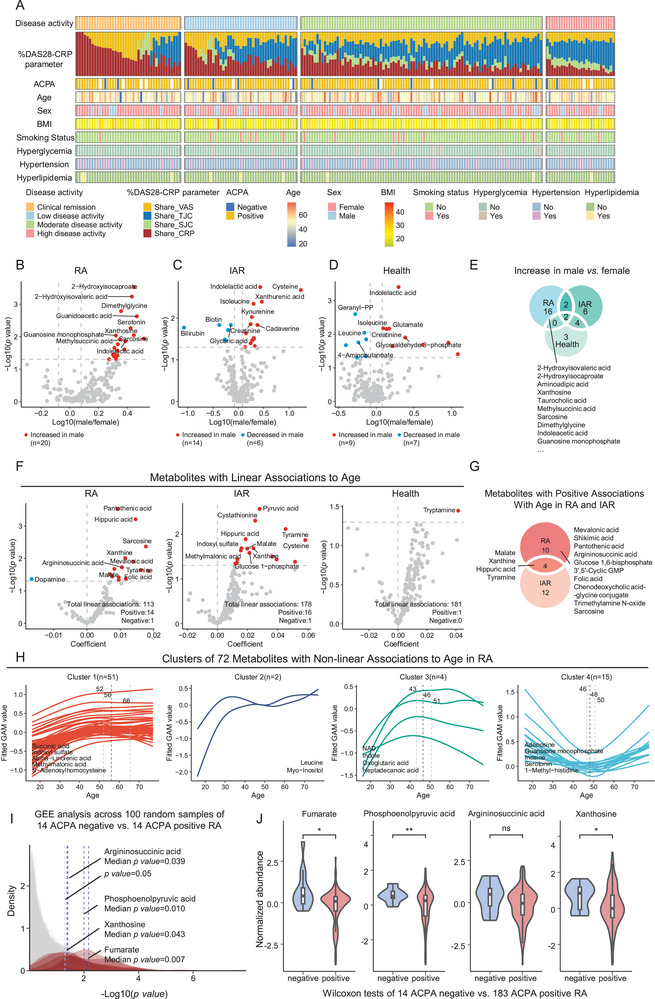
Influences of sex, age, and ACPA status on metabolomic profiles in RA. A) Heatmap presenting baseline clinical data for 209 RA patients, including disease activity groups (clinical remission: DAS28‐CRP < 2.6; low disease activity: 2.6 ≤ DAS28‐CRP ≤ 3.2; moderate disease activity: 3.2 < DAS28‐CRP ≤ 5.1; high disease activity: DAS28‐CRP > 5.1), the percentage composition of DAS28‐CRP parameters: shared_TJC28 = $\frac{0.56\times \sqrt{TJC28}}{DAS28\_CRP-0.96}$, shared_SJC28 = $\frac{0.28\times \sqrt{SJC28}}{DAS28\_CRP-0.96}$, shared_CRP = $\frac{0.36\times \ln\;(\mathrm{CRP}+1)}{DAS28\_CRP-0.96}$, shared_VAS =$\frac{0.014\times (\mathrm{VAS})}{DAS28\_CRP-0.96}\;$, anti‐citrullinated protein autoantibodies (ACPA), age, sex, BMI, smoking and commodities status, with blanks indicating missing data. B–D) Volcano plots illustrating sex‐associated metabolite differences within RA (B), IAR (C), and health (D). Red dots indicate metabolites significantly higher in males, whereas blue dots represent metabolites significantly lower in males (|fold change|>1.2, *p* < 0.05, Wilcoxon test). E) Venn diagram showing overlapping significant metabolites with increased levels in males compared to females across RA, IAR, and health. F) Volcano plots showing metabolites with linear age associations based on GAMs model in RA (left), IAR (middle), and health (right). Red dots indicate metabolites significantly increased with age (coefficient > 0, *p* < 0.05, two‐sided *t*‐test). G) Venn diagram showing overlapping metabolites significantly increased with age between RA and IAR. H) Line plots of age‐associated metabolites with non‐linear trends, clustered by their fitted trajectories from GAMs. I) Density plot from 100 iterations of GEE model regression (adjusted for age, sex, and CRP) comparing 14 randomly selected ACPA positive RA patients with 14 ACPA negative RA patients, significance of regression coefficients was determined by two‐sided *t*‐test. J) Violin plots with embedded boxplots (the median and interquartile range) displaying the results of the Wilcoxon test for differential metabolites between 183 ACPA‐positive RA and 14 ACPA‐negative RA, with ^*^ *p* < 0.05 and ^**^ *p* < 0.01, “ns” indicates not significant.

Sex, age, and ACPA status influence disease prevalence, comorbidities, joints involvement, and disease duration in RA, and their impact on metabolism has also been reported.^[^ ^18^, ^19^, ^20^ ^]^ However, previous studies have rarely conducted a systematic investigation of the associations between these clinical variables and metabolic profiles in RA. To address this gap, we performed corresponding analyses utilizing metabolomic data from 209 baseline RA patients. Most of them had moderate activity according to european league against rheumatism (EULAR) classification standard (clinical remission: DAS28‐CRP < 2.6; low disease activity: 2.6 ≤ DAS28‐CRP ≤ 3.2; moderate disease activity: 3.2 < DAS28‐CRP ≤ 5.1; high disease activity: DAS28‐CRP > 5.1) (**Figure** **3**A).

We analyzed the correlation between metabolites and sex in RA. A total of 20 sex‐associated metabolites were identified in RA, all of which were elevated in males (Figure 3B; Table S3A, Supporting Information). Notably, 16 of these 20 metabolites were positively associated with male sex only in the RA group, including glycine methylation products such as sarcosine and dimethylglycine, suggesting that aberrant methylation contributes to sex differences in RA (Figure 3C–E; Table S3A, Supporting Information). Only 2 metabolites showed shared correlation to male among RA, IAR, and healthy individuals, suggesting that sex‐associated metabolic differences in RA were primarily driven by disease mechanisms rather than baseline physiological sex differences.

We next assessed the impact of age on RA metabolism, adjusting for disease duration to distinguish effects of aging from cumulative disease burden. For each metabolite, both linear and non‐linear models were fitted using generalized additive models (GAMs). A metabolite was considered suitable for linear modeling if the estimated degrees of freedom (edf) from the non‐linear GAMs was less than 1.5, or if the akaike information criterion (AIC) of the non‐linear model was higher than that of the linear model. In RA patients, 113 metabolites exhibited linear age associations, with 14 showing significant positive correlations and only one showing a negative correlation (Figure 3F; Table S3B, Supporting Information). Most age‐associated metabolites in RA showed no significant correlation with age in IAR or health, indicating that these age‐related changes—particularly methylation‐related metabolites such as sarcosine and folic acid—are specific to RA (Figure 3G). Beyond linear trends, 72 metabolites showed non‐linear associations with age and were grouped into four trajectory‐based clusters (Figure 3H; Table S3C, Supporting Information). Cluster 1 (*n* = 51) showed gradual increases, leveling off around age 60. Cluster 2 (*n* = 2), including leucine and myo‐inositol, rose sharply before age 40, stabilized, then increased again. Cluster 3 (*n* = 4) steadily increased from age 20, peaking around 40–50 before declining. Cluster 4 (*n* = 15) showed a biphasic pattern—initial decline (20–50 years), followed by a gradual rise (50–70 years).

Furthermore, we explored the correlation between metabolites and ACPA status. To compare the metabolic differences between the ACPA subsets, we randomly selected 14 ACPA‐positive RA in each iteration, comparing their metabolites to the 14 ACPA‐negative RA using GEE model adjusted for CRP, age, and sex, and repeated this process 100 times. As a result, fumarate, phosphoenolpyruvic acid (PEP), xanthosine, and argininosuccinic acid showed median *p* values below 0.05 and were elevated in ACPA‐negative RA (Figure 3I; Table S3D, Supporting Information). Except for argininosuccinic acid, the other three also exhibited significance in the overall comparison between 183 ACPA‐positive and 14 ACPA‐negative patients using Wilcoxon test (Figure 3J). Fumarate supports metabolism of innate immune cells, while argininosuccinic acid contributes to its production and promotes oxidative stress.^[^ ^21^ ^]^ These metabolic alterations may partially contribute to the pathological mechanisms underlying the distinct clinical manifestations observed between ACPA‐positive and ACPA‐negative RA subgroups.

### 2.4 Metabolomic Shifts Link to RA Development and Severity

**Figure 4:**
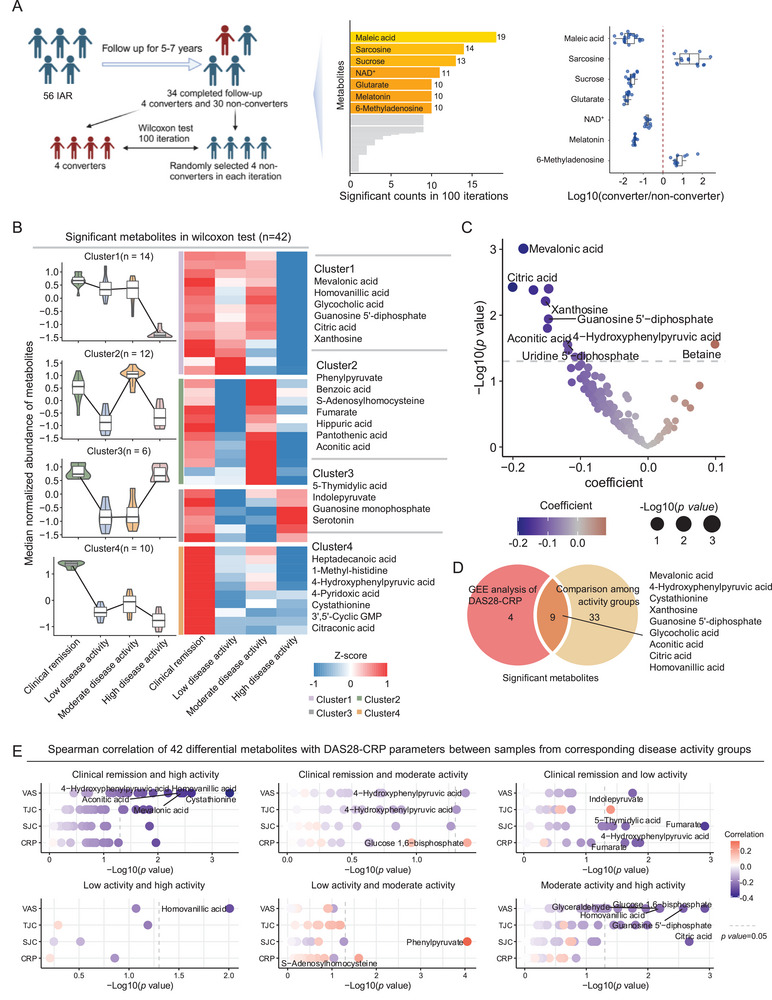
Investigation into the metabolites linked to development and disease activity in RA. A) Left: Analytical framework for assessing metabolite differences between converters (developed into RA) and non‐converters (not developed into RA) in IAR. Middle: Bar plot showing the significant counts of metabolites (*p* < 0.05, Wilcoxon test) between 4 converters and 4 randomly selected non‐converters across 100 iterations, with 7 top metabolites exceeding 10 significant counts highlighted. Right: Boxplots (the median and interquartile range) showing the distribution of log10(fold change) across significant iterations for the 7 top metabolites, with the red dashed line indicating log10(0). B) Left: Violin plots with embedded boxplots (median and interquartile range) depicting cluster‐specific metabolite level variations, with significance (*p* < 0.05, Wilcoxon test) determined via pairwise disease activity group comparisons. Middle: Heatmap displaying the median levels (scaled) of metabolites in distinct clusters. Right: Representative metabolites for each cluster. C) GEE model analysis of DAS28‐CRP and metabolites (adjusted for age, sex, BMI, smoking status, and comorbidities), with red indicating positively related (coefficient > 0, *p* < 0.05, two‐sided *t*‐test) and blue indicating negatively related (coefficient < 0, *p* < 0.05, two‐sided *t*‐test). D) Venn diagram showing the overlap of significant metabolites identified through the Wilcoxon test and GEE model analysis. E) The dot plot displays the correlation of significant metabolites from comparisons between disease activity groups with four DAS28‐CRP parameters based on Spearman analysis. Gray dashed lines denote the statistical significance threshold (*p* = 0.05).

To identify metabolites associated with the development of RA, we compared individuals who developed RA (converters) with those who did not (non‐converters) in a cohort of 34 IAR followed for 5 to 7 years, of whom 4 progressed to RA (Table S4A, Supporting Information). Given the small number of converters, we randomly selected 4 non‐converters in each iteration and used the Wilcoxon test to compare their metabolites with the 4 converters, repeating this 100 times (**Figure** **4**A). Seven metabolites had *p*<0.05 in over 10 iterations (Table S4B, Supporting Information). Maleic acid, sucrose, glutarate, NAD^+^, and melatonin showed lower levels in converters. Notably, sarcosine and 6‐methyladenosine were elevated in converters, indicating abnormal methylation activity during disease progression.

To identify metabolic alterations associated with baseline DAS28‐CRP levels in RA, we compared metabolite differences across disease activity groups using the Wilcoxon test. The heatmap and box plots display the levels of 42 metabolites that are significant between any two groups in the Wilcoxon test, organized into four clusters based on their changing patterns (Figure 4B; Table S4C, Supporting Information): Most of the differential metabolites belonged to Cluster 1 and Cluster 4, showing general decrease from low to high disease activity, including mevalonic acid, homovanillic acid, glycocholic acid and citric acid, which were also negatively associated with DAS28‐CRP in GEE model after adjusting for clinical confounding (Figure 4C,D; Table S4D, Supporting Information). Cluster 2 demonstrated a wave‐like pattern of changes across the activity groups, encompassing metabolites such as aconitic acid, phenylpyruvate, and benzoic acid. Cluster 3 displayed an initial slight decrease, followed by an increase at high activity levels, such as guanosine monophosphate. Notably, only betaine, a methyl donor, showed a positive association in the GEE model, suggesting a link between aberrant methylation and disease activity (Figure 4C).

In the higher activity groups, swollen joint count (SJC) and tender joint count (TJC) had a greater weighting in the disease score, while CRP and visual analog scale (VAS) contributed more to the lower activity groups (Figure 3A). Given this differential contribution of DAS28‐CRP parameters across activity levels, we next investigated which parameters were most closely associated with the observed metabolic differences. For each metabolite showing significant differences between any two disease activity groups (as determined by the Wilcoxon test), Spearman correlation analysis was performed between the metabolite levels and four DAS28‐CRP parameters using samples from the corresponding groups (Figure 4E; Table S4E, Supporting Information). We found that most of the differential metabolites between the high disease activity group and others were primarily associated with VAS. Specifically, anti‐inflammatory molecules such as mevalonic acid and homovanillic acid showed lower levels in the high activity group compared to the remission group, and all were negatively correlated with VAS (upper left panel in Figure 4E). Glucose 1,6‐bisphosphate and guanosine 5′‐diphosphate, which were more abundant in the high activity group than in the moderate activity group, also showed negative correlations with VAS (lower right panel in Figure 4E). In contrast, metabolite differences involving the low activity group were primarily associated with SJC: The pro‐inflammatory metabolite phenylpyruvate was elevated in the moderate activity group relative to the low activity group and positively correlated with SJC (lower middle panel in Figure 4E). These findings suggest that modulating these metabolites may help relieve joint pain in patients with high disease activity and reduce joint swelling in lower activity levels.

### 2.5 Metabolome Reveals Predictive Insights for csDMARDs Response Through Machine Learning and MTX‐Based Metabolic Modulation

MTX‐based regimens are the standard treatment for RA, yet patients' responses to various drug combinations can differ significantly. A comprehensive analysis of treatment outcomes from longitudinal studies remains lacking. To fill this gap, we next explored the predictive potential of metabolite levels, combined with clinical indicators, to assess the therapeutic response of RA. Evaluations based on the EULAR criteria were conducted after a treatment period of more than three months. We concentrated on the MTX+LEF (*n* = 73) and MTX+HCQ (*n* = 47) groups, as their sample sizes allowed for reliable statistical analysis (**Table** **2**). While age and sex showed no differences, higher VAS, TJC, and SJC were linked to better responses in MTX+LEF, and higher VAS and TJC were associated with better responses in MTX+HCQ (Figure S1A,B, Supporting Information).

**Table 2:** Clinical attributes of the MTX+HCQ and MTX+LEF treatment group.

Baseline Clinical Information	MTX+HCQ (N = 47)	MTX+LEF (N = 73)
Female, n (%)	39(83%)	57(78.1%)
Age, years	51(19–75)	53(16–77)
Body Mass Index, kg m^−2^	22.60(15.94–31.13)	22.27(14.38–29.41)
Time from symptom onset to diagnosis, months	25 (0–420)	23 (2–372)
Time from diagnosis to sampling, months	70 (0–492)	62 (0–360)
Duration, months	94 (6–498)	85 (6–378)
Baseline DAS28‐CRP	3.70 (1.74–6.59)	3.77 (1.44–8.39)
Disease Activity Level, n (%)		
Clinical Remission	8 (17%)	10 (13.7%)
Low Disease Activity	11 (23.4%)	19 (26%)
Moderate Disease Activity	22 (46.8%)	33 (45.2%)
High Disease Activity	6 (12.8%)	11 (15.1%)
CRP, mg L^−1^	14.54 (1–197)	16.36 (1–138)
Tender Joints In 28‐Joint Count	6 (0–28)	6 (0–28)
Swollen Joints In 28‐Joint Count	2 (0–10)	2 (0–23)
Visual Analog Scale	45 (0–100)	47 (0–100)
Health Assessment Questionnaire	7.09 (0–40)	7.82 (0–42)
Smokers, n (%)	5 (10.6%)	9 (12.3%)
With comorbidity, n (%)		
Hypertension	10 (21.2%)	12 (16.4%)
Hyperlipidemia	4 (8.5%)	3 (4.1%)
Hyperglycemia	5 (10.6%)	4 (5.4%)

**Figure 5:**
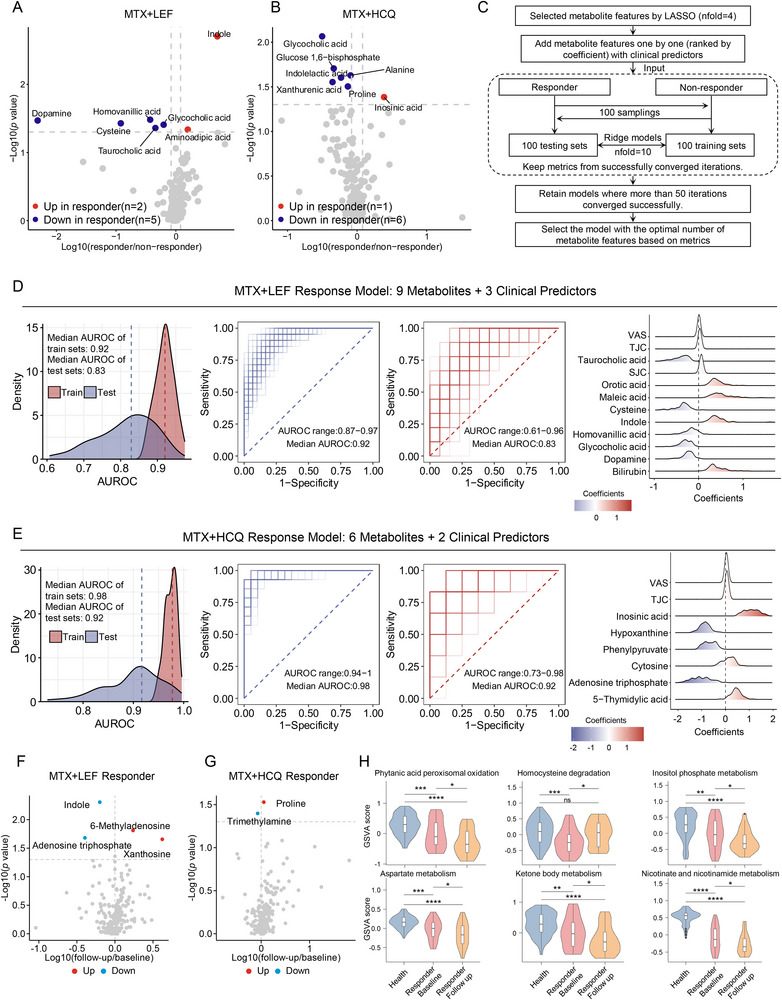
Predicting drug response and investigating drug effects. A) Volcano plots showing the differential metabolites between responders (*n* = 30) and non‐responders (*n* = 43) in the MTX+LEF group identified by the Wilcoxon test (*p* < 0.05). B) Volcano plots illustrating the differential metabolites between responders (*n* = 20) and non‐responders (*n* = 27) in the MTX+HCQ group by Wilcoxon test (*p* < 0.05). C) Flowchart of constructing a machine learning model to predict drug response. D) Performance evaluation of the machine learning model for predicting response to MTX+LEF based on clinical and metabolic features across converged iterations. (Left) Distribution of area under the receiver operating characteristic curve (AUROC) values for train (red) and test (blue) sets. (Middle) ROC curves for the train set. (Right) ROC curves for the test set. (Far Right) Coefficient contributions of features to the predictive model. E) Performance evaluation of the machine learning model for predicting response to MTX+HCQ based on clinical and metabolic features across converged iterations. (Left) Distribution of AUROC values for train (red) and test (blue) sets. (Middle) ROC curves for the train set. (Right) ROC curves for the test set. (Far Right) Coefficient contributions of features to the predictive model. F) Volcano plots showing differential metabolites between baseline and follow‐up samples of responders in the MTX+LEF (*n* = 30) by paired Wilcoxon test (*p* < 0.05). G) Volcano plots showing differential metabolites between baseline and follow‐up samples of responders in the MTX+HCQ (*n* = 20) by paired Wilcoxon test (*p* < 0.05). H) Violin plots with embedded boxplots (the median and interquartile range) depicting significantly enriched pathways among health (blue, *n* = 99), baseline (red, *n* = 30), and follow‐up samples (yellow, *n* = 30) of responders in MTX+LEF groups. Significance was calculated by the Wilcoxon test using GSVA scores. Significance levels: ^*^ *p* < 0.05, ^**^ *p* < 0.01, ^***^ *p* < 0.001, “ns” indicates not significant.

We identified differential metabolites between responders and non‐responders. Based on EULAR response criteria, there were 30 responders and 43 non‐responders in the MTX+LEF group. In the MTX+HCQ group, there were 20 responders and 27 non‐responders. In the MTX+LEF group, responders showed elevated levels of indole and aminoadipic acid, while dopamine, homovanillic acid, and other metabolites were lower than in non‐responders (**Figure** **5**A; Table S5A, Supporting Information). In the MTX+HCQ group, responders had higher levels of inosinic acid, along with decreased glycocholic acid, glucose 1,6‐bisphosphate, and so on (Figure 5B; Table S5A, Supporting Information).

To build a model for predicting treatment response, we used the least absolute shrinkage and selection operator (LASSO) to select metabolite features and incorporated them with differential clinical indicators for model construction. While increasing the number of features may enhance model performance, excessive feature inclusion can lead to convergence failures. To balance these factors, metabolites were ranked by the absolute values of their LASSO‐derived coefficients and incrementally added to the model to determine the optimal number of features (Table S5B, Supporting Information). Responders and non‐responders were randomly divided into training and testing sets at a 7:3 ratio. A ridge regression model was implemented and iterated 100 times, with 10‐fold cross‐validation performed in each iteration to identify the optimal regularization parameter (Figure 5C). To evaluate model robustness, area under the receiver operating characteristic curve (AUROC) values from each iteration were retained. For MTX+LEF, the AUROC of both training and testing sets steadily increased with the number of metabolite features, and all nine candidate features could be included while still maintaining over 50 successful convergences. In contrast, for MTX+HCQ, AUROC improved with more features, but convergence fell below 50 iterations beyond six features; therefore, the top six features were selected for the final model (Figure S2A,B, Supporting Information).

In both the MTX+LEF and MTX+HCQ groups, incorporating metabolite features enhanced the predictive accuracy of models based solely on clinical parameters. In the MTX+LEF group, the model's median AUROC on the test set improved from 0.63 to 0.83 with the inclusion of nine metabolite features. The median integrated discrimination improvement (IDI) values remained positive in both training (0.282) and test sets (0.207), supporting the added discriminative value of metabolite features over clinical predictors alone (Figure 5D; Figure S2C,D, Table S5C–E, Supporting Information). Similarly, in the MTX+HCQ group, the median AUROC increased from 0.77 to 0.92 with the inclusion of six metabolite features. Median IDI values were 0.312 in the training set and 0.221 in the test set, indicating improved discriminative performance (Figure 5E; Figure S2E,F, Table S5F–H, Supporting Information).

MTX‐based therapy has an under‐optimal efficacy. To understand the metabolic basis of the efficacy of these MTX‐based treatments, we analyzed metabolome changes using pre‐ and post‐treatment data from responders to the MTX+LEF and MTX+HCQ regimens. Our analysis showed that after MTX+LEF treatment, levels of 6‐methyladenosine and xanthosine increased, while adenosine triphosphate and indole decreased. In the MTX+HCQ group, proline increased, and trimethylamine decreased following therapy (Figure 5F,G; Table S5I, Supporting Information). Moving beyond the single‐molecule level, we also examined the whole metabolic pathways modified by drugs via GSVA analysis. Several pathways exhibited differences between baseline samples and both health and follow‐up samples. Among these, homocysteine degradation demonstrated a notable drug effect, with its levels being lower in baseline samples than in health but restored by MTX+LEF treatment. In contrast, other pathways such as nicotinate, nicotinamide metabolism, and aspartate metabolism were further reduced by the drugs, despite already being lower in baseline samples, highlighting the MTX+LEF uncovering effect (Figure 5H; Table S5J, Supporting Information). The MTX+HCQ modification was not so obvious that only ketone body metabolism was significantly reduced after therapy.

## 3 Discussion

This study applied broadly targeted metabolomic profiling to systematically characterize metabolic alterations across the RA disease continuum. We identified a broad spectrum of metabolic alterations associated with RA development, disease activity, age, sex, and ACPA status, with metabolites related to methylation and redox imbalance emerging as prominent features. Additionally, based on data from 3–6‐month follow‐up of RA patients under MTX+LEF or MTX+HCQ therapy, we developed predictive models integrating clinical indicators and metabolic features, achieving robust performance with AUROC values of 0.83 for MTX+LEF and 0.92 for MTX+HCQ.

Targeted metabolomics is a hypothesis‐driven approach that quantitatively analyzes predefined metabolites within specific pathways, offering greater sensitivity and selectivity than non‐targeted metabolomics.^[^ ^22^, ^23^ ^]^ Previous studies have primarily employed non‐targeted metabolomics followed by targeted biomarker validation, which is useful for discovery but limited in capturing pathway‐level metabolic changes.^[^ ^24^ ^]^ While some studies have adopted targeted approaches, these were often restricted by small cohort sizes or a limited metabolite panel.^[^ ^25^ ^]^ Additionally, previous studies often lacked rigorous medication control during RA enrollment, raising concerns about potential confounding. In contrast, our study used a targeted metabolomics strategy encompassing 238 metabolites across 42 pathways, previously applied in other inflammation studies.^[^ ^14^, ^15^, ^16^ ^]^ Notably, all 209 baseline RA patients had not received any DMARDs within six months prior to enrollment. We observed metabolic changes that were in line with past studies in RA, such as decreased citric acid, cysteine, glutamine,^[^ ^26^ ^]^ and bilirubin,^[^ ^27^ ^]^ and increased succinic acid, glyceric acid,^[^ ^28^ ^]^ and trimethylamine N‐oxide (TMAO).^[^ ^29^ ^]^ Novel metabolite change patterns were also observed, including decreased hypoxanthine and increased BCAAs levels, which contrast with findings from other RA metabolomic studies and may reflect differences in medication control across cohorts.^[^ ^28^, ^30^, ^31^ ^]^ The metabolic shift from oxidative phosphorylation to glycolysis is a well‐established feature of RA pathogenesis, and such a shift has been associated with reduced hypoxanthine levels;^[^ ^32^ ^]^ BCAAs are typically elevated in obesity and type 2 diabetes and have been implicated in promoting inflammation.^[^ ^33^, ^34^ ^]^ These lines of evidence may support the plausibility of our findings. In addition, we found that metabolic alterations—such as increased levels of AA, 12‐HETE, and 13‐HODE—were already present in IAR, potentially serving as early diagnostic indicators. Notably, these changes have not been reported in previous studies.^[^ ^35^, ^36^ ^]^

Sex influences disease prevalence, duration, and comorbidity profiles in RA. Although females have a higher incidence of RA, disease activity in early RA is generally comparable between sexes, while males tend to present with more severe joint damage and a higher incidence of cardiovascular disease.^[^ ^37^, ^38^ ^]^ In our study, several metabolites were elevated both in RA patients compared to healthy individuals and in males compared to females. Among them, 2‐hydroxyisovaleric acid has been proposed as a biomarker for early RA detection.^[^ ^39^ ^]^ The elevation of dimethylglycine, which has been linked to reduced skeletal muscle mass in RA, and serotonin, known to impair osteoblast function and disrupt bone metabolism, may help explain the greater extent of bone erosion observed in male patients.^[^ ^40^, ^41^ ^]^ Additionally, increased levels of dimethylglycine and N8‐acetylspermidine—respectively associated with coronary heart disease and ischemic cardiomyopathy, may contribute to the higher cardiovascular risk reported in males with RA.^[^ ^42^, ^43^ ^]^ Age also contributes to RA heterogeneity, as elderly‐onset RA is characterized by acute systemic symptoms, involvement of larger joints, poorer functional outcomes, and a greater burden of comorbidities.^[^ ^44^, ^45^ ^]^ In our study, several age‐associated metabolites showing a positive linear correlation were identified. TMAO has been shown to promote vascular inflammation and contribute to the development of atherosclerosis.^[^ ^46^ ^]^ Tyramine may lead to lipid accumulation and hepatic inflammation.^[^ ^47^ ^]^ The upregulation of xanthine may reflect purine metabolic dysregulation in CD4⁺ T cells under stress conditions.^[^ ^48^ ^]^ Notably, these sex‐ and age‐associated metabolic alterations were not present in healthy individuals, suggesting that such changes are RA‐specific and may underlie sex‐ and age‐related disease heterogeneity.

ACPA‐negative RA is often characterized by higher disease activity and reduced treatment responsiveness to B‐cell depletion therapies or csDMARDs compared to ACPA‐positive RA.^[^ ^49^, ^50^ ^]^ In ACPA‐positive RA, adaptive immune cells—particularly T and B cells—play central roles in disease progression, whereas ACPA‐negative RA is characterized by heightened activity of innate immune cells.^[^ ^51^ ^]^ In our study, fumarate exhibited the most significant increase in ACPA‐negative RA than ACPA‐positive RA. Fumarate, involved in the TCA cycle, has been shown to promote innate immune responses by modulating the epigenome,^[^ ^52^ ^]^ may represent a promising therapeutic target for improving outcomes in ACPA‐negative RA, particularly in patients who exhibit poor responses to B‐cell depletion therapies.

We identified several metabolites associated with disease activity. In addition to previously reported metabolites negatively associated with disease activity—such as tryptophan, bilirubin,^[^ ^27^ ^]^ and citrate^[^ ^53^ ^]^—we identified novel ones, including homovanillic acid, an anti‐inflammatory metabolite,^[^ ^54^ ^]^ and aconitic acid, which has been linked to pain relief states.^[^ ^55^ ^]^ Both were significantly reduced in the high activity group compared to the remission group. These findings suggest that they may serve as potential therapeutic targets for alleviating pain in patients with high disease activity, thereby contributing to improved symptom control and quality of life in RA.

Pursuant to established guidelines, csDMARDs are recognized as the foundational approach for managing RA, despite encountering limitations due to adverse effects and less than optimal efficacy in drug response. Previous studies on predictive metabolites for drug response either involved MTX‐based combinations (regardless of the accompanying drugs) or focused on biologic treatments.^[^ ^56^ ^]^ In contrast, we conducted separate analyses for the two most common RA treatment regimens in China: MTX+LEF and MTX+HCQ. Our development of drug response predictive models with optimal performance held the adding value of metabolic features to refine personalized treatment strategies.

Altered DNA methylation has been implicated in RA pathogenesis.^[^ ^57^ ^]^ In our study, we further identified that dysregulated levels of methylation‐related metabolites are associated with RA development, disease activity, age, sex, and ACPA status. Specifically, we observed significantly reduced levels of SAM (the sole methyl group donor involved in the methylation of DNA and RNA) in RA compared to IAR and health. This reduction may reflect abnormal DNA methylation activity and immune aging in RA.^[^ ^58^ ^]^ Other methyl donors, including folate, betaine, and methionine, were elevated in RA; folate increased with age, and betaine was positively associated with disease activity. Methylation products such as sarcosine increased with age, males, and notably in IAR converters. The decrease in SAM and increase in sarcosine may result from the upregulation of glycine N‐methyltransferase under chronic inflammation.^[^ ^59^ ^]^ Other methylation products, such as dimethylglycine (elevated in males) and 6‐methyladenosine (elevated in IAR versus health and IAR converters), further suggest that dysregulated methylation contributes to RA pathogenesis and clinical heterogeneity.

SAM is a precursor for GSH synthesis, and its reduction can lead to lipid peroxidation and increased oxidative stress.^[^ ^60^ ^]^ In our study, we also observed elevated oxidative stress in RA. Specifically, antioxidant metabolites such as NAD^+^, GSH, and GSSG were negatively associated with disease status, showing reduced levels in both RA and IAR; notably, NAD^+^ was also significantly decreased in IAR converters. Argininosuccinic acid, elevated with age and negative ACPA status, has been reported to reduce GSH and enhance lipid peroxidation.^[^ ^61^ ^]^ Cystine and cysteine, key building blocks of GSH, were reduced in both RA and IAR, with cysteine serving as a negative predictor of response to MTX+LEF treatment.

Although this study enriched our understanding of both pathogenic mechanisms and pharmacological interventions in RA, it is imperative to acknowledge its limitations. Our emphasis on plasma metabolomics within the circulatory system may have neglected nuances inherent to the synovium, a pivotal site in RA pathology. Due to the small number of converters (*n* = 4), the predictive analysis for RA development is exploratory in nature. Although we applied a bootstrapping‐based resampling strategy to mitigate class imbalance, the limited sample size constrains statistical power and generalizability. Larger prospective cohorts are needed to validate these potential biomarkers. Moreover, while informative, our analysis of drug responsiveness would benefit from additional validation in a novel cohort. These considerations delineate pathways for future research to refine and broaden our comprehension of this intricate spectrum of autoimmune diseases.

## 4 Experimental Section

### Clinical Sample Collection—Cohort Establishment

The study collected plasma samples from 99 healthy individuals, 56 IAR, and 209 patients with RA at West China Hospital, Sichuan University. These were approved by the Research Ethics Committee of West China Hospital, Sichuan University (Permission number: 2021(790)), and informed consent was obtained from all participants.

### Clinical Sample Collection—The Inclusion Criteria Included

Patients were diagnosed with RA according to the 2010 American College of Rheumatology (ACR)/ EULAR criteria. All RA patients were DMARDs‐free for at least six months prior to enrollment. All enrolled RA patients were required to exhibit initial symptom onset at ≥16 years of age. Cases with documented symptom onset prior to 16 years were excluded to ensure cohort homogeneity with adult‐onset RA. The EULAR defines IAR as those meeting one or more of the following criteria: a) genetic predisposition to RA, b) environmental factors linked to RA, c) systemic autoimmunity related to RA, d) symptoms without clinical arthritis, or e) unclassified arthritis. In our study, IAR participants were specifically categorized under phase “c”; they were all ACPA‐positive individuals without any symptoms.^[^ ^62^ ^]^ Healthy individuals consisted of age‐ and sex‐matched individuals without any history or clinical signs of autoimmune or rheumatic diseases. The exclusion criteria include a history of cancer, organ transplantation, or other infectious diseases. About classification criteria, ACPA serostatus was determined using Elecsys anti‐cyclic citrullinated peptide electrochemiluminescence immunoassay on Cobas e 801 analyzer (Roche Diagnostics, Mannheim, Germany), with results categorized as positive (≥17.0 U ml^−1^) or negative (<17.0 U ml^−1^).^[^ ^63^ ^]^

### Clinical Sample Collection—Follow‐Up Design and Sample Collection

A longitudinal observational protocol was implemented for participants with RA and IAR. Standardized follow‐up assessments for RA were conducted at 3–6‐month intervals following treatment initiation with csDMARDs, comprising systematic evaluations of clinical indicators (e.g., joint swelling, tenderness, and CRP) alongside biological specimen acquisition. Blood collection followed standard venipuncture procedures using anticoagulant tubes. Plasma samples were obtained after centrifugation and stored at −80 °C until batch testing. For IAR, we conducted telephone interviews with each participant to ascertain whether they had been diagnosed with RA by a hospital and to record the specific date of diagnosis if applicable.

### Targeted Metabolomics—Metabolite Extraction

The sample preparation was performed as previously described.^[^ ^15^, ^16^ ^]^ First, all the plasma samples were randomized and were thawed by transferring them from −80 °C storage to a 4 °C refrigerator. Following vortex mixing, 20 µL aliquots were extracted from each of the first 160 samples, pooled, and divided into QCs samples (50 µL each). Subsequently, 50 µL of plasma samples was transferred to another tube and mixed with 250 µL of pre‐cooled Spike MeOH containing isotopically labeled internal standards (120.89 µm 13C6‐D‐glucose and 23.12 µm 13C5‐15N‐L‐glutamate). The mixture was thoroughly vortexed at 1500 rpm for 2 min at 4 °C (MSC‐100, Allsheng, Hangzhou, China). It was then incubated at −20 °C for 30 min. Ultrasonication was subsequently performed in an ice‐water bath for 10 min (SB‐2200‐DT, Scientz, Ningbo, China). After ultrasonication, the mixture was centrifuged at 13,000 rpm (Thermo Micro 17R, Thermo Fisher, MA, USA) for 20 min at 4 °C. A volume of 150 µL of the supernatant was collected, vacuum‐dried, and reconstituted in 0.2 mL of HILIC buffer (internal standards: 14.74 µm 13C9‐15N‐L‐tyrosine and 43.52 µm 13C‐L‐lactate in 30% mobile phase A and 70% mobile phase B). Following centrifugation, 100 µL of the supernatant was transferred to sampling vials. Aliquots of 4 and 15 µL were injected for LC‐MS/MS analysis in positive and negative ion modes, respectively. Robust QCs were ensured through the calculation of CV for isotope‐labeled internal standards added during sample preparation across all samples.

### Targeted Metabolomics—LC‐MS/MS‐based Targeted Metabolomics

Metabolite profiling was conducted using a SCIEX ExionLC UHPLC system coupled with a Triple Quad 5500+ mass spectrometer (AB Sciex, Framingham, MA) operating in MRM mode.^[^ ^15^, ^16^ ^]^ Chromatographic separation was achieved on an ACQUITY UPLC BEH Amide column (100 × 2.1 mm, 1.7 µm, Waters, Milford, MA, USA) maintained at 40 °C. The mobile phase consisted of two components: A (90% H2O, 10% acetonitrile, 10 mm ammonium acetate, and 0.2% acetic acid) and B (90% acetonitrile, 10% H2O, 10 mm ammonium acetate, and 0.2% acetic acid), delivered at a flow rate of 0.3 mL min^−1^ for gradient elution. The gradient program was as follows: 0–1.5 min, 90% B; 1.5–5 min, linear decrease to 45% B; 5–10 min, hold at 45% B; 10–12 min, increase to 90% B; 12–25 min, hold at 90% B. QCs samples were interspersed every 10 injections to ensure system stability. These QCs samples were also utilized for normalizing the samples.

The MS parameters were configured as follows: for positive ion mode, curtain gas (CUR): 35.0, collision gas (CAD): 8.0, ion spray voltage (IS): 5500 V; for negative ion mode, curtain gas (CUR): 35.0, collision gas (CAD): 8.0, ion spray voltage (IS): ‐4500 V. The temperature was set to 550.0 °C, ion source gas 1 (GS1): 55.0, and ion source gas 2 (GS2): 55.0. MRM mode‐based broadly targeted metabolomics was employed to detect metabolites of interest according to our previously published protocol.^[^ ^64^ ^]^ A total of 238 MRM ion transitions corresponding to 42 metabolic pathways were selected for targeted analysis.^[^ ^15^ ^]^ Data acquisition was performed using Analyst 1.7.2 software, and data processing was carried out using SCIEX OS (version 2.1.6.59781) software (AB Sciex, Framingham, MA).

### Biomarker Identification—Normalization of Metabolite Data

Normalization was achieved by the following steps: Dividing the level of each metabolite by the average value of adjacent QCs samples. A cross‐sample total sum correction was conducted across all metabolites. Metabolites with a CV greater than 0.30 across QCs samples and those with more than 80% missing values across all samples were removed. The remaining missing values were substituted with one‐fifth of the minimum positive value for each variable. Subsequently, a log10 transformation was applied, and each variable was mean‐centered and divided by its standard deviation.

### Biomarker Identification—Bioinformatics Analysis

PLS‐DA was performed using the “mixOmics” R package (ncomp = 10) to identify the overall metabolic differences among RA, IAR, and health. The correlations between QCs samples and between the metabolites and DAS28‐CRP parameters were calculated using Spearman's correlation coefficient. GSVA was performed using the “GSVA” R package. For regression‐based analysis, we applied marginal GEE models, using the geeglm() function from “geepack” R package. The statistical significance of each regression coefficient was assessed using a two‐sided *t*‐test. This included evaluating relationships between metabolites (or GSVA scores) and clinical groups, age, and DAS28‐CRP. For metabolite and clinical groups, each metabolite was modeled as:

Normalized metabolite abundance = clinical group + confounding factors (sex + age + BMI + smoking status + hypertension + hyperlipidemia + hyperglycemia)

For metabolite and DAS28‐CRP, each metabolite was modeled as:

Normalized metabolite abundance = DAS28‐CRP + confounding factors (sex + age + BMI + smoking status + hypertension + hyperlipidemia + hyperglycemia)

Specifically, for the analysis of age‐related effects, we first evaluated whether each metabolite exhibited a linear relationship with age by GAMs from “mgcv” R package, with disease duration adjusted as a covariate. For metabolites that were adequately modeled using a linear approach, defined by an edf less than 1.5 or a lower AIC in the linear model compared to the non‐linear GAMs, we applied the following linear regression formula:

Normalized metabolite abundance = Age + s(duration)

For metabolites that were more appropriately modeled by non‐linear relationships, the following GAMs formula was used:

Normalized metabolite abundance = s(Age) + s(duration)

Furthermore, for metabolites described by non‐linear age associations, we clustered their predicted trajectories and visualized the results.

Between group comparisons (e.g., sex, disease activity levels, response groups) were assessed using the Wilcoxon rank‐sum test (“wilcox.test()” function in base R).

The proportions of each parameter's contribution to the total DAS28‐CRP were calculated by transforming each parameter according to the formula:^[^ ^65^ ^]^

DAS28‐CRP $=0.56\times \sqrt{\mathrm{TJC}28}$+0.28×$\sqrt{\mathrm{SJC}28}$ +0.36×ln (CRP+1) + 0.014×(VAS)+0.96.

Specifically, the contributions in Figure 3A were as follows:

shared_TJC28 = $\frac{0.56\times \sqrt{TJC28}}{DAS28\_CRP-0.96}$, shared_SJC28 = $\frac{0.28\times \sqrt{SJC28}}{DAS28\_CRP-0.96}$,

shared_ CRP = $\frac{0.36\times \ln\;(\mathrm{CRP}+1)}{DAS28\_CRP-0.96}$, shared_ VAS = $\frac{0.014\times (\mathrm{VAS})}{DAS28\_CRP-0.96}\;$.

To compare subgroups with unequal sizes (e.g., 183 ACPA‐positive versus 14 ACPA‐negative RA patients; 52 non‐converters versus 4 converters in IAR), we applied a bootstrapping‐based resampling strategy. In each of 100 iterations, a random subset of ACPA‐positive or non‐converters samples was selected to match the smaller group. For ACPA status, a marginal GEE model adjusted for sex, age, and CRP was applied in each iteration. All pathway enrichment analyses were performed based on the SMPDB. All visualizations were generated using the “ggplot2” package. The classification and distribution of the 185 metabolites by superclass were annotated according to the Human Metabolome Database.

### Machine Learning for Treatment Response

To predict patient response to MTX+LEF combination therapy, we developed a binary classification model using both clinical indicators and metabolite features. The analysis was conducted in R v4.3.1. Response classification followed the EULAR response criteria:^[^ ^66^ ^]^ Good response was characterized by a DAS28‐CRP improvement of >1.2 and a present DAS28‐CRP ≤3.2. Moderate response was defined as a DAS28‐CRP improvement >1.2 with a present DAS28‐CRP >3.2, or a DAS28‐CRP improvement between 0.6 and ≤1.2 with a present DAS28‐CRP ≤5.1. No response was defined as a DAS28‐CRP improvement between 0.6 and ≤1.2 with a present DAS28‐CRP >5.1, or a DAS28‐CRP improvement ≤0.6 regardless of the present DAS28‐CRP. Patients classified as having good or moderate responses were grouped as responders, and the rest as non‐responders.

### Machine Learning for Treatment Response—Feature Selection

For metabolite features, LASSO regression (“glmnet” package, alpha = 1) was applied separately for each drug regimen. To ensure a sufficient sample size for model convergence, 4‐fold cross‐validation was used in both cases. This approach identified 9 candidate features for the MTX+LEF group and 30 for the MTX+HCQ group. To determine the optimal model, metabolite features were ranked by the absolute value of their LASSO coefficients. Features were then iteratively added to clinical predictors to build ridge models, and model performance metrics were used to select the optimal number of features. Wilcoxon tests were used to evaluate clinical variables, including DAS28‐CRP components (TJC, SJC, CRP, VAS), sex, and age. Variables that showed significant differences between responders and non‐responders (*p* < 0.05) were retained as clinical predictors.

### Machine Learning for Treatment Response—Model Construction

Ridge regression (alpha = 0) was applied using the “glmnet” package to construct binary classification models, with metabolite features sequentially added based on their ranking by absolute LASSO coefficients, enabling robust comparison across models with increasing feature numbers. In each of 100 iterations, 70% of responder and non‐responder samples were randomly selected to form the training set, and the remaining 30% were used as the test set. The training set was used to fit a ridge regression model via cv.glmnet() with 10‐fold cross‐validation to determine the optimal regularization parameter (lambda). The fitted model was applied to both training and test sets to generate predicted probabilities. Model performance was evaluated on both datasets, including AUROC, sensitivity, specificity, positive predictive value (PPV), and negative predictive value (NPV), using the multipleROC() function. An optimal cutoff was derived from the multipleROC() function to calculate accuracy. Model coefficients from each iteration were extracted and stored for downstream interpretation. With increasing feature numbers, convergence failures occurred more frequently, due to the imbalance between model complexity and sample size. Iterations that failed to converge were excluded from further analysis to ensure model robustness. In the final evaluation, only models with more than 50 successful iterations were retained. The model with the optimal number of features was then selected based on the median AUROC across iterations.

To assess the added predictive value of metabolite features beyond clinical predictors alone, IDI analysis was performed on the final selected model. In each iteration, predictions from the clinical‐only model and the clinical plus metabolite model were compared using the reclassification() function, based on predicted probabilities generated from both the training and testing sets.

### Machine Learning for Treatment Response—Visualization and Interpretation

Density plots of AUROC values across iterations were generated using “ggplot2” package, and their distributions were summarized by median and range. ROC curves for all iterations (train/test) were visualized using the “plot_ROC()” function. Coefficient distributions across 100 models were visualized using “ggridges” package. Dot and error bar plots were generated to visualize IDI values and their 95% confidence intervals across iterations, highlighting statistically significant improvements (*p* < 0.05).

### Statistical Analysis

Continuous variables in Tables 1 and 2 were presented as mean (range), and categorical variables as counts (percentage). Boxplots in the figures display medians and interquartile ranges. Group comparisons were performed using the Wilcoxon test (non‐parametric data), GEE or GAMs models (adjusted for covariates). The significance of coefficients was performed using two‐sided *t*‐tests. The significance of detection frequency differences of metabolites across groups was assessed using Fisher's exact test (for expected counts <5) or the chi‐square test (otherwise). The cluster of disease activity or age‐associated metabolites was performed using Ward's minimum variance method. Statistical significance was defined as *p* < 0.05, *p* <0.01, and *p* < 0.001, denoted by ^*^, ^**^, and ^***^, respectively. “ns” indicates not significant. All analyses were performed in R version 4.3.1, and visualizations were generated using the “ggplot2” package.

### Code Availability

The source code, including differential computation, feature selection, prediction models, and plotting, was publicly available on GitHub: https://github.com/alice370/metabolism.git

## Conflict of Interest

The authors declare no conflict of interest.

## Author Contributions

C.Z., J.X., S.H., and Q.N. contributed equally to this work. C.Z., J.X., S.H., and Q.N. performed conceptualization, data curation, formal analysis, investigation, visualization, methodology, wrote the original draft, and edited the final manuscript. Y.L., X.G., and Q.Z. performed investigation, methodology, and resources. H.H., R.S., Y.L., and L.Y. performed the investigation and methodology. Z.X. and N.J. performed data curation and methodology. T.C. performed supervision, acquired funding acquisition and project administration. L.D., F.Z., L.M., I.G., and R.H. reviewed and edited the final manuscript. B.Y., D.D., and Y.Z. performed conceptualization, resources, data curation, formal analysis, supervision, acquired funding acquisition, project administration, reviewed and edited the final manuscript.

## Supporting information



Supporting Information

Supporting Information

Supporting Information

Supporting Information

Supporting Information

Supporting Information

## Data Availability

The mass spectrometry metabolomic data have been submitted to the Metabolomics Workbench (https://www.metabolomicsworkbench.org/) under the Project ID PR001976 (doi: 10.21228/M89J06).
