# Supplementary material for: A Longitudinal Study Reveals Metabolomic Markers for Individuals at Risk, Disease Severity, and Treatment Response in Rheumatoid Arthritis
Source: Adv Sci (Weinh). 2025 Aug 13;12(38):e04414. doi: 10.1002/advs.202504414 (PMC12520550; doi:10.1002/advs.202504414)
Supplement: Supplementary file 6 — Supporting Information [file ADVS-12-e04414-s006.pptx]

## Slide 1
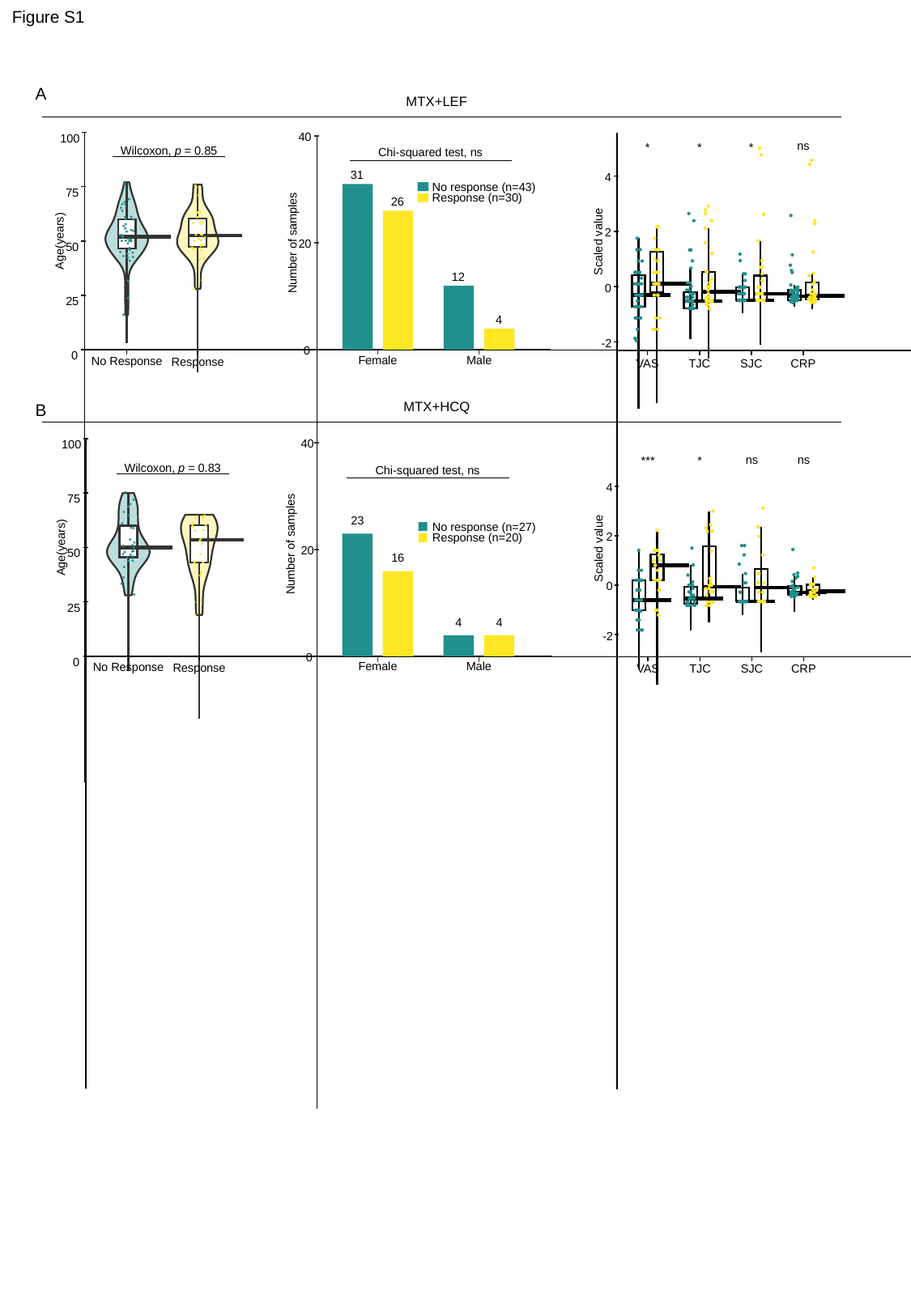

Figure S1
A
MTX+LEF
100
Wilcoxon, p = 0.85
75
Age(years)
50
25
0
No Response
Response
ns
*
*
*
4
2
Scaled value
0
-2
VAS
TJC
SJC
CRP
40
Chi-squared test, ns
31
No response (n=43)
Response (n=30)
26
Number of samples
20
12
4
0
Female
Male
MTX+HCQ
B
100
Wilcoxon, p = 0.83
75
Age(years)
50
25
0
No Response
Response
ns
ns
***
*
4
2
Scaled value
0
-2
VAS
TJC
SJC
CRP
40
Chi-squared test, ns
No response (n=27)
Response (n=20)
23
Number of samples
20
16
4
4
0
Female
Male

## Slide 2
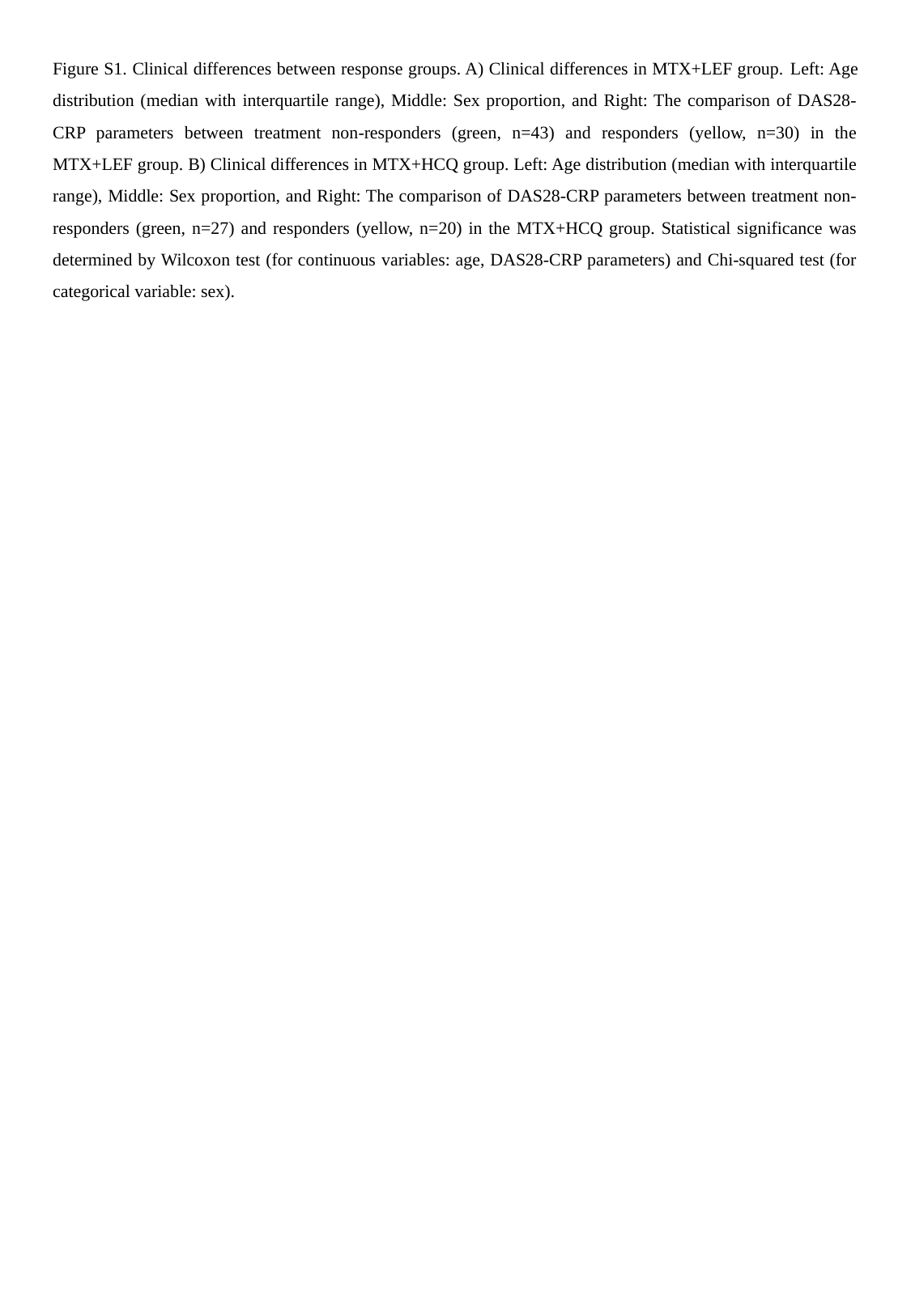

Figure S1. Clinical differences between response groups. A) Clinical differences in MTX+LEF group. Left: Age distribution (median with interquartile range), Middle: Sex proportion, and Right: The comparison of DAS28-CRP parameters between treatment non-responders (green, n=43) and responders (yellow, n=30) in the MTX+LEF group. B) Clinical differences in MTX+HCQ group. Left: Age distribution (median with interquartile range), Middle: Sex proportion, and Right: The comparison of DAS28-CRP parameters between treatment non-responders (green, n=27) and responders (yellow, n=20) in the MTX+HCQ group. Statistical significance was determined by Wilcoxon test (for continuous variables: age, DAS28-CRP parameters) and Chi-squared test (for categorical variable: sex).

## Slide 3
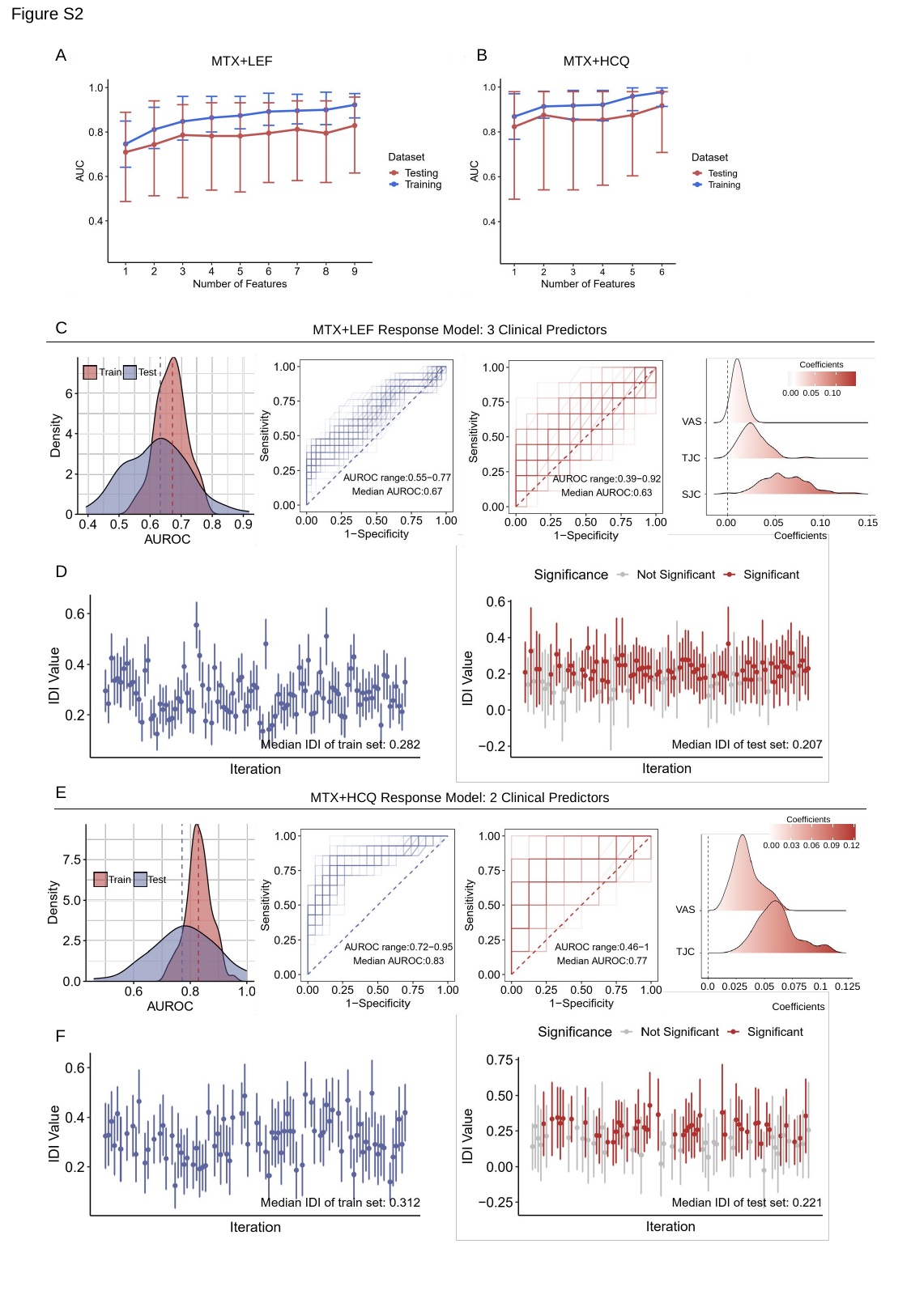

Figure S2
A
B
MTX+LEF
MTX+HCQ
C
MTX+LEF Response Model: 3 Clinical Predictors
Coefficients
0.00
0.05
0.10
Train
Test
VAS
TJC
SJC
0.00
0.05
0.10
0.15
Coefficients
D
E
MTX+HCQ Response Model: 2 Clinical Predictors
Coefficients
0.00
0.03
0.06
0.09
0.12
Train
Test
VAS
TJC
0.0
0.025
0.05
0.075
0.1
0.125
Coefficients
F

## Slide 4
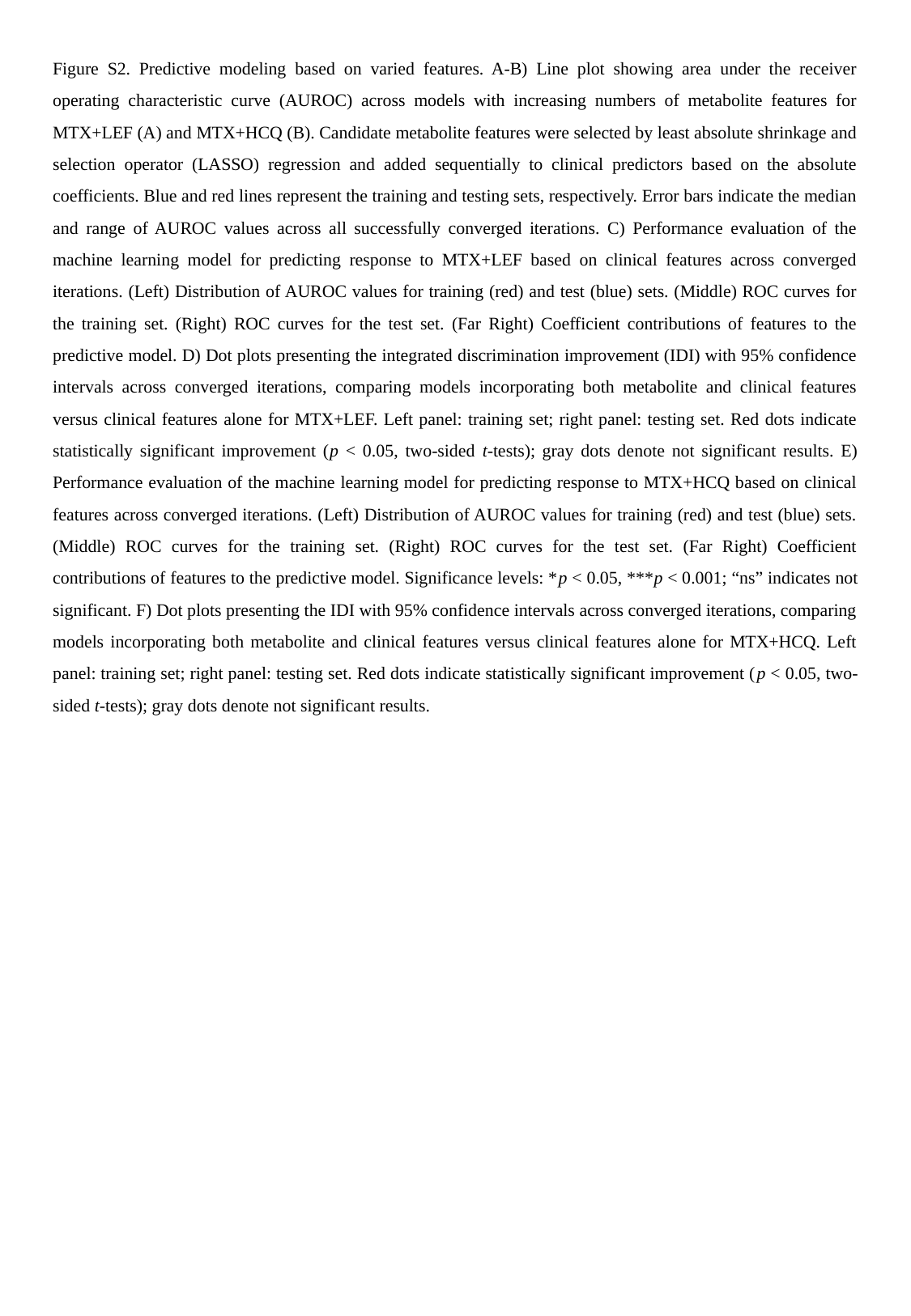

Figure S2. Predictive modeling based on varied features. A-B) Line plot showing area under the receiver operating characteristic curve (AUROC) across models with increasing numbers of metabolite features for MTX+LEF (A) and MTX+HCQ (B). Candidate metabolite features were selected by least absolute shrinkage and selection operator (LASSO) regression and added sequentially to clinical predictors based on the absolute coefficients. Blue and red lines represent the training and testing sets, respectively. Error bars indicate the median and range of AUROC values across all successfully converged iterations. C) Performance evaluation of the machine learning model for predicting response to MTX+LEF based on clinical features across converged iterations. (Left) Distribution of AUROC values for training (red) and test (blue) sets. (Middle) ROC curves for the training set. (Right) ROC curves for the test set. (Far Right) Coefficient contributions of features to the predictive model. D) Dot plots presenting the integrated discrimination improvement (IDI) with 95% confidence intervals across converged iterations, comparing models incorporating both metabolite and clinical features versus clinical features alone for MTX+LEF. Left panel: training set; right panel: testing set. Red dots indicate statistically significant improvement (p < 0.05, two-sided t-tests); gray dots denote not significant results. E) Performance evaluation of the machine learning model for predicting response to MTX+HCQ based on clinical features across converged iterations. (Left) Distribution of AUROC values for training (red) and test (blue) sets. (Middle) ROC curves for the training set. (Right) ROC curves for the test set. (Far Right) Coefficient contributions of features to the predictive model. Significance levels: *p < 0.05, ***p < 0.001; “ns” indicates not significant. F) Dot plots presenting the IDI with 95% confidence intervals across converged iterations, comparing models incorporating both metabolite and clinical features versus clinical features alone for MTX+HCQ. Left panel: training set; right panel: testing set. Red dots indicate statistically significant improvement (p < 0.05, two-sided t-tests); gray dots denote not significant results.
